# *QuickStats:* Percentage* of Adults Aged 18–64 Years Who Used Telemedicine in the Past 12 Months,^†^ by Sex and Health Insurance Coverage^§^ — National Health Interview Survey, United States 2021^¶^

**DOI:** 10.15585/mmwr.mm7209a5

**Published:** 2023-03-03

**Figure:**
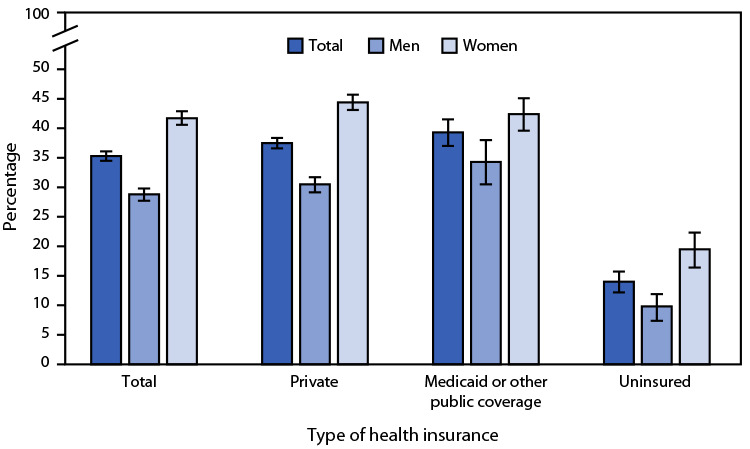
Overall, in 2021, 35.3% of adults aged 18–64 years had a telemedicine visit with a health care professional in the past 12 months. The percentage was higher among women than men overall (41.7% versus 28.8%). Women were also more likely than were men to have had a telemedicine visit among those with private health insurance (44.4% versus 30.5%), Medicaid or other public coverage (42.4% versus 34.3%), and those who were uninsured (19.5% versus 9.8%). Adults with private health insurance (37.5%) or Medicaid or other public coverage (39.3%) were more likely to use telemedicine compared with uninsured adults (14.0%), and this pattern was seen for women and men.

**Source:** National Center for Health Statistics, National Health Interview Survey, 2021. https://www.cdc.gov/nchs/nhis.htm

